# Detection of highly pathogenic zoonotic influenza virus H5N6 by reverse-transcriptase quantitative polymerase chain reaction

**DOI:** 10.1186/s12985-015-0250-3

**Published:** 2015-02-08

**Authors:** Hans G Heine, Adam J Foord, Jianning Wang, Stacey Valdeter, Som Walker, Chris Morrissy, Frank YK Wong, Brian Meehan

**Affiliations:** CSIRO Australian Animal Health Laboratory, 5 Portarlington Road, Geelong, VIC 3220 Australia

**Keywords:** Influenza virus, Avian influenza, Reassortant virus, Molecular diagnosis, H5N6, Clade 2.3.4.4, PCR

## Abstract

**Background:**

Variant high pathogenicity avian influenza (HPAI) H5 viruses have recently emerged as a result of reassortment of the H5 haemagglutinin (HA) gene with different neuraminidase (NA) genes, including NA1, NA2, NA5, NA6 and NA8. These viruses form a newly proposed HA clade 2.3.4.4 (previously provisionally referred to as clade 2.3.4.6), and have been implicated in disease outbreaks in poultry in China, South Korea, Laos, Japan and Vietnam and a human fatality in China. There is real concern that this new clade may be wide spread and not readily identified using existing diagnostic algorithms.

**Findings:**

Fluorescent probe based reverse-transcriptase quantitative polymerase chain reaction (RT-qPCR) assays were developed to facilitate the identification of novel clade 2.3.4.4 viruses of H5N6 subtype emerging in Asia. Assays were aimed at the haemagglutinin (HA) gene for clade identification and at the NA gene to identify N6. The HA assay employing a minor groove binder (MGB) probe was able to detect and differentiate A/duck/Laos/XBY004/2014(H5N6) and related influenza A(H5N6) virus isolates belonging to the proposed clade 2.3.4.4 from other H5 HPAI viruses. In addition, an Eurasian N6 assay was able to differentiate N6 from other NA subtypes.

**Conclusions:**

Laos influenza A(H5N6) virus representative of proposed clade 2.3.4.4, was detected and differentiated from viruses in other H5N1 clades using a clade-specific HA RT-qPCR assay whereas the N6-NA subtype was determined by an Eurasian N6 RT-qPCR assay. Such a clade-specific assay would be of particular value for surveillance and in diagnostic laboratories where sequencing is not readily available.

## Findings

HPAI viruses continue to evolve and pose a threat to animal and human health. Recent HPAI H5 virus variants of different NA subtypes have been implicated in disease outbreaks in poultry in China (H5N1, H5N2, H5N5, H5N6, H5N8) , South Korea (H5N8), Japan (H5N8), Laos (H5N8) and Vietnam (H5N1, H5N6) and an H5N6 subtype virus caused a human fatality in China [1-3]. These variants have previously been referred to as “clade 2.3.4.6” viruses and originate from H5N1 clade 2.3.4 viruses [4,5]. However, the WHO/OIE/FAO H5N1 Evolution Working Group recently designated that these viruses fall into a new clade 2.3.4.4 and not 2.3.4.6 [6]. Of particular significance, these emergent H5 viruses have a propensity for reassortment of the HA gene with different NA subtypes, including NA1, NA2, NA5, NA6, NA8 [7]. Accordingly there is concern that this clade may be spreading more widely [8], and although these variant strains are being detected by diagnostic RT-qPCR assays in current use, they cannot be readily distinguished from other H5N1 HPAI viruses.

Molecular identification of this new clade currently requires conventional RT-PCR amplification of the HA gene followed by nucleotide sequence determination. Likewise, identification of the N subtype requires nucleotide sequence determination following conventional RT-PCR of the NA gene or multiple N6 subtype-specific RT-qPCR assays. Here we report the development of RT-qPCR assays to facilitate the identification of A/duck/Laos/XBY004/2014(H5N6) [1] and other closely related H5N6 HPAI viruses that may have zoonotic potential [3].

Primers and probes for HA and NA specific RT-qPCR assays were designed to complement sequences of the A/duck/Laos/XBY004/2014(H5N6) and related viruses. Other HA sequences that belong to the new proposed clade 2.3.4.4 (Table 1) were identified using Basic Local Alignment Search Tool (BLAST) [9] analysis of A/duck/Laos/XBY004/2014(H5N6) against the GenBank nucleotide sequence database (nr/nt) [10]. Subsequently, sequence alignments were performed using Geneious software (Version 7.1.6, Biomatters). Potential primer and probe regions distinctive of proposed clade 2.3.4.4 viruses were identified from these alignments and candidate primers and probes assessed using the Primer Express 3.0.1 program (Applied Biosystems). The clade specific HA probes contained the codon for amino acid substitution 273H that has been identified as one of the distinguishing amino acids for clade 2.3.4.4 viruses [4]. Two candidate probes were designed using black hole quencher 1 (BHQ1) and minor groove binder (MGB) chemistry, respectively. Primer and probe sequences and optimized concentrations of the new and reference assays are listed in Table 2. These assays were developed using thermocycling conditions compatible with the reference avian influenza (AI) type A and the H5 duplex RT-qPCR reference assays [11], (diagnostic manual, CSIRO Australian Animal Health Laboratory, unpublished). Reactions were performed using AgPath-ID One-Step RT-PCR Kit (Ambion - Applied Biosystems) on an AB7500 Fast instrument using the thermal cycle: 1 cycle of 45°C 10 min, 95°C 10 min followed by 45 cycles of 95°C 15 sec, 60°C 45 sec. For interpretation of results samples producing a cycle threshold (C_T_) less than 40 were considered positive, C_T_ values 40.1 to 45 were considered indeterminate.

**Table 1:** **HPAI H5 proposed clade 2.3.4.4 strains containing different N types**

**Influenza A virus**	**Subtype**	**Accession**
A/wild duck/Shandong/628/2011	H5N1	JX534565
A/chicken/China/AH/2012	H5N1	KC631941
A/muscovy duck/Vietnam/LBM631/2014	H5N1	AB979455
A/muscovy duck/Vietnam/LBM635/2014	H5N1	AB979487
A/muscovy duck/Vietnam/LBM636/2014	H5N1	AB979495
A/duck/Vietnam/LBM638/2014	H5N1	AB979503
A/duck/Eastern China/1111/2011	H5N2	JQ041401
A/goose/Eastern China/1112/2011	H5N2	JQ041402
A/duck/Jiangsu/m234/2012	H5N2	JX507355
A/duck/Guangdong/wy11/2008	H5N5	CY091627
A/duck/Guangdong/wy19/2008	H5N5	CY091635
A/environment/Zhenjiang/C13/2013	H5N6	KJ938658
A/duck/Guangdong/GD01/2014	H5N6	KJ754145
A/duck/Lao/XBY004/2014	H5N6	KM496977
A/duck/Jiangsu/k1203/2010	H5N8	JQ973694
A/duck/Zhejiang/W24/2013	H5N8	KJ476669
A/duck/Zhejiang/6D18/2013	H5N8	KJ476670
A/breeder duck/Korea/Gochang1/2014	H5N8	KJ413834
A/broiler duck/Korea/Buan2/2014	H5N8	KJ413842
A/baikal teal/Korea/Donglim3/2014	H5N8	KJ413850
A/chicken/Kumamoto/1-7/2014	H5N8	AB932556

The influenza A virus, segment 4 hemagglutinin (HA) gene sequences were identified by BLASTn analysis of HA sequence from A/duck/Laos/XBY004/2014(H5N6) (GenBank accession performed on 2^nd^ September, 2014). The listed strains share 100% identity to the target region of probes D-720 PRB and D-724 PRB (MGB) of the proposed H5 clade 2.3.4.4 specific qRT-PCR assays. The H5 strains are sorted by N subtype N1, N2, N5, N6, and N8.

**Table 2:** **Primer and probe oligonucleotides for qRT-PCR assays used in this study**

**Assay/Oligo**	**Sequence (5′ to 3′)**	**Final conc. (μM)**
**AI Type A**		
IVA D161M	AGATGAGYCTTCTAACCGAGGTCG	0.9
IVA D162M1	TGCAAAAACATCYTCAAGTCTCTG	0.225
IVA D162M2	TGCAAACACATCYTCAAGTCTCTG	0.225
IVA D162M3	TGCAAAGACATCYTCAAGTCTCTG	0.225
IVA D162M4	TGCAAATACATCYTCAAGTCTCTG	0.225
IVA MA	(FAM)-TCAGGCCCCCTCAAAGCCGA-(TAMRA)	0.25
**H5 duplex**		
IVA D148H5	AAACAGAGAGGAAATAAGTGGAGTAAAATT	0.675
IVA D149H5	AAAGATAGACCAGCTACCATGATTGC	0.675
IVA H5A	(FAM)-TCAACAGTGGCGAGTTCCCTAGCA-(TAMRA)	0.3
IVA D204f	ATGGCTCCTCGGRAACCC	0.675
IVA D205r	TTYTCCACTATGTAAGACCATTCCG	0.675
IVA D215P	(FAM)-ATGTGTGACGAATTCMT-(MGB-NFQ)	0.3
**H5 2.3.4.4-BHQ1**		
D-718 FWD	CAAGAAAGGGGACTCAACAATTATG	0.3
D-719 REV	TGAGAGGGTGTATATTGTGGAATGG	0.9
D-720 PRB	(FAM)-TTGACATTTGGTGTTGCAGTG**G**CCATA-(BHQ1)	0.25
	[TTGACATTTGGTGTTGCAGTG**C**CCATA*]	
**H5 2.3.4.4-MGB**		
D-718 FWD	CAAGAAAGGGGACTCAACAATTATG	0.3
D-719 REV	TGAGAGGGTGTATATTGTGGAATGG	0.9
D-724 PRB (MGB)	(FAM)-ATGG**C**CACTGCAAC-(MGB-NFQ)	0.25
	[ATGG**G**CACTGCAAC*]	
**AI N6**		
D-721 FWD	CCCCACCAATGGGAACTG	0.9
D-722 REV	TCTAGGAATGCAAACCCTTTTACC	0.3
D-723 PRB	(FAM)-CCAATAACAGGAGGGAGCCCAGACCC-(BHQ1)	0.25

*H5N6 2.3.4.4 virus with a single nucleotide mismatch in the TaqMan probe sequences has also been recently detected. Accordingly, to ensure greatest potential future utility additional probe sequences containing the single nucleotide substitution are given in bold.

The assays used in this study were:

- **AI Type A** (generic). This is a generic assay targeting the matrix gene to detect influenza A viruses from different animal hosts, including avian, equine and/or other species. This assay is in routine use for AI diagnosis at the CSIRO Australian Animal Health Laboratory and Australian state veterinary diagnostic laboratories.
- **H5 duplex** (generic H5). This is a generic assay to detect H5 subtypes, consisting of two sets of primers and probes targeting two different regions (C-terminus and N-terminus) of the H5 HA gene.
- **H5 2.3.4.4-BHQ1** (candidate clade specific). This assay was designed specifically for detection of new proposed HA clade 2.3.4.4 strains and utilizes a BHQ1 probe.
- **H5 2.3.4.4-MGB** (candidate clade specific). This assay was designed specifically for detection of new proposed HA clade 2.3.4.4 strains and utilizes a MGB probe. The same primers were used for both H5 2.3.4.4 assays.
- **AI N6**. This assay design was based on A/duck/Laos/XBY004/2014(H5N6) to detect Asian lineage N6-NA of novel A(H5N6) viruses, however it is not specific for all N6 viruses.

Reaction efficiencies and limit of detection (LOD) of the RT-qPCR assays were determined for the A/duck/Laos/XBY004/2014(H5N6) isolate (5 × 10^9^/ml median embryo infective dose) using a serial 10 fold dilution of template and assays were performed in triplicate. The reaction efficiencies were 80.5, 92.0, 87.0, 91.2 and 86.3% for the AI type A, H5 duplex, H5 2.3.4.6-BHQ1, H5 2.3.4.6-MGB and AI N6, respectively. The LOD for all assays was observed at the 10^−7^ dilution, equivalent to approximately five median embryo infective doses for the given template and displaying the last positive C_T_ values between 37.9 and 38.5 (data not shown).

The capability of the candidate RT-qPCR assays to differentiate proposed clade 2.3.4.4 viruses from other H5 HPAI viruses was evaluated using A/duck/Laos/XBY004/2014(H5N6) as a representative of clade 2.3.4.4 viruses in comparison with other relevant AI reference virus strains (Table 3).

**Table 3:** **Comparison of RT-qPCR assays**

**Sample description**	**RT-qPCR assays**				
	**AI type A (generic)**	**H5 duplex (generic H5)**	**H5 2.3.4.4-BHQ1**	**H5 2.3.4.4-MGB**	**AI N6**
A/shearwater/Australia/75(H5N3)	**22.9**	**25.3**	U	U	U
A/Chicken/Myanmar/295/2010(H5N1); clade 2.3.2.1	**20.8**	**22.8**	U	U	U
A/Duck/VN/2064/13(H5N1); clade 2.3.2.1	**19.8**	**20.8**	U	U	U
A/Swallow/Vietnam/4696/2013(H5N1); clade 2.3.2.1	**17.7**	**19.3**	U	U	U
A/Ch/LaoPDR/LK001/2010(H5N1); clade 2.3.4.1	**17.6**	**21.3**	U	U	U
A/Ch/LaoPDR/LK002/2010(H5N1); clade 2.3.4.1	**17.7**	**21.6**	U	U	U
A/Duck/VN/4804/13(H5N1); clade 1.1	**22.7**	**24.2**	**28.8**	U	44.8/U
A/Chicken/Indonesia/Subang/2007(H5N1); clade 2.1.3	**23.2**	**24.6**	**30.2**	U	U
A/Chicken/Laos/Xaythani/26/2006(H5N1); clade 2.3.4	**24.0**	**27.4**	**31.7**	U	U
A/Avian/Myanmar/182/2010(H5N1); clade 2.3.4.2	**20.7**	**23.5**	**26.8**	U	42.3/U
**A/duck/Lao/XBY004/2014(H5N6); clade 2.3.4.4**	**20.8**	**24.9**	**22.7**	**18.2**	**22.1**
A/MALLARD/GURJEV/244/82(H14N6)	**21.5**	U	U	U	**23.0**
A/silver gull/Tasmania/349/2006(H13N6)	**27.1**	U	U	U	U
A/gull/Maryland/704/1977(H13N6)	**15.7**	U	U	U	40.2/U
No template (negative control)	U	U	U	U	U

AI reference assays and candidate clade-specific and N6 assays were compared using selected AI strains.

U = Undetected. Results are displayed as averaged C_T_ values of two replicates. Assay results considered positive are indicated by C_T_ values in bold. Strain A/duck/Lao/XBY004/2014(H5N6) representative of proposed clade 2.3.4.4 is indicated in bold.

Laos A(H5N6) isolates were detected by the current RT-qPCR AI type A and H5 duplex assays however, only the H5 2.3.4.4-MGB RT-qPCR was able to distinguish these novel A(H5N6) viruses belonging to the proposed clade 2.3.4.4 strain from other AI viruses including A(H5N1). In contrast, the H5 2.3.4.4-BHQ1 RT-qPCR detected A/duck/Laos/XBY004/2014(H5N6) but was not able to differentiate from some other A(H5N1) clades as detailed in Table 3. The AI N6 qRT-PCT assay differentiated the Laos H5N6 subtype virus from H5N1 strains, thus providing useful rapid diagnostic differentiation from A(H5N1) that co-circulate in domestic poultry in the region (Table 3).

This evaluation was based on A/duck/Laos/XBY004/2014(H5N6) that was available for testing in our laboratory. However, *in silico* sequence analysis indicated that other N6 and non-N6 viruses in proposed clade 2.3.4.4 were identical in the sequences of the HA primer and probe target region (Table 1), but distinguished from other A(H5N1) by at least one nucleotide change in the probe region. As experimentally proven in this study, the mismatches in the MGB probe target region were sufficient to provide the required analytical specificity for clade 2.3.4.4 virus differentiation.

Accordingly, this sensitive and clade-specific assay will be of particular value for surveillance programs and in diagnostic laboratories where routine DNA sequencing is not readily performed or available.

### Availability of supporting data

The data sets supporting the results of this article are included within the article.

## Abbreviations

AI: Avian influenza
BHQ1: Black hole quencher 1
HA: Haemagglutinin
HPAI: High pathogenicity avian influenza
LOD: Limit of detection
MGB: Minor groove binder
NA: Neuraminidase
RT-qPCR: Reverse-transcriptase quantitative polymerase chain reaction
